# *Wolbachia* in guilds of *Anastrepha* fruit flies (Tephritidae) and parasitoid wasps (Braconidae)

**DOI:** 10.1590/1678-4685-GMB-2016-0075

**Published:** 2016-09-19

**Authors:** Rodrigo O Mascarenhas, Leandro F Prezotto, André Luiz P Perondini, Celso Luiz Marino, Denise Selivon

**Affiliations:** 1 Departamento de Genética e Biologia Evolutiva, Instituto de Biociências, Universidade de São Paulo (USP), São Paulo, SP, Brazil; 2 Departamento de Genética, Instituto de Biociências, Universidade Estadual Paulista (UNESP), Botucatu, SP, Brazil

**Keywords:** Bacteria, fruit flies, horizontal transmission, *wsp* gene, recombination

## Abstract

The endosymbiont *Wolbachia* is efficiently transmitted from females to their progenies, but horizontal transmission between different taxa is also known to occur. Aiming to determine if horizontal transmission might have occurred between *Anastrepha* fruit flies and associated braconid wasps, infection by *Wolbachia* was screened by amplification of a fragment of the *wsp* gene. Eight species of the genus *Anastrepha* were analyzed, from which six species of associated parasitoid wasps were recovered. The endosymbiont was found in seven *Anastrepha* species and in five species of braconids. The WSP Typing methodology detected eight *wsp* alleles belonging to *Wolbachia* supergroup A. Three were already known and five were new ones, among which four were found to be putative recombinant haplotypes. Two samples of *Anastrepha obliqua* and one sample of *Doryctobracon brasiliensis* showed multiple infection. Single infection by *Wolbachia* was found in the majority of samples. The distribution of *Wolbachia* harboring distinct alleles differed significantly between fruit flies and wasps. However, in nine samples of fruit flies and associated wasps, *Wolbachia* harbored the same *wsp* allele. These congruences suggest that horizontal transfer of *Wolbachia* might have occurred in the communities of fruit flies and their braconid parasitoids.

## Introduction

The endosymbiotic bacteria *Wolbachia* (alphaproteobacteria; Rickettsiales) is an intracellular parasite. It has been associated with the manipulation of its host's reproduction by induction of several phenotypes, such as cytoplasmic incompatibility (CI) in several insect species, parthenogenesis in Hymenoptera, feminization of genetic males, and male killing in Coleoptera, Lepidoptera, Diptera and Pseudoscorpiones (Werren, 1997; Bourtzis and O'Neill, 1998; Bourtzis *et al.*, 2003; Werren *et al.*, 2008). However, the bacteria may be beneficial to their hosts by interfering positively in several fitness components of males and females. In such cases, the relationships between *Wolbachia* and their hosts evolved from a status of parasitism to mutualistic relationships (Werren *et al.*, 2008; Serbus *et al.*, 2008; Saridaki and Bourtzis, 2010). Previous data have indicated that species infection rates were variable but could account for the infection of 40 to 70% of arthropod species (Werren and Windsor, 2000; Jeyaprakash and Hoy, 2000; Hilgenboecker *et al.*, 2008; Zug and Hammerstein, 2012).

*Wolbachia* are found dispersed in various tissues of the hosts, and their presence in the female germ line assures a highly efficient maternal transmission (Werren, 1997; Dobson *et al.*, 1999). Although the infection is usually pervasive in populations, even if it started with few infected females, it was argued that vertical transmission alone does not explain the large distribution of the bacteria among arthropods. Moreover, phylogenies of *Wolbachia* are usually incongruent with phylogenies of their hosts. Hence, horizontal transmission was assumed as a possible mechanism promoting the spread of the bacteria among taxa of related organisms, as well as among those showing close relationships, like prey-predator, parasite-host, and parasitoid-hosts (O'Neill *et al.*, 1992; Werren *et al.*, 1995, 2008; Vavre *et al.*, 1999; Noda *et al.*, 2001; Dedeine *et al.*, 2005; Baldo *et al.*, 2006a, 2008; Stahlhut *et al.*, 2010; Pattabhiramaiah *et al.*, 2011; Le Clec'h *et al.*, 2013). Other ways of horizontal transmission were found between species of herbivorous insects that acquire *Wolbachia* strains by ingesting tissues of the host plants contaminated with the bacteria (Kittayapong *et al.*, 2003; Sintupachee *et al.*, 2006; Yang *et al.*, 2013), or by contact of haemolymph through wounds in the host's bodies (Rigaud and Juchault, 1995). Horizontal transmission was also considered the route of infection by multiple *Wolbachia* strains, as is frequently observed in many species of Coleoptera, Diptera, Hymenoptera and Lepidoptera (Werren *et al.*, 1995; Jamnongluk *et al.*, 2002; Rokas *et al.*, 2002; Reuter and Keller, 2003; Hiroki *et al.*, 2004; Schuler *et al.*, 2011; Yang *et al.*, 2012, 2013; Augustinos *et al.*, 2014).

Experimentally, natural transmission of bacteria was found between a non-infected parasitoid (*Leptopilina boulardi*) that acquired some *Wolbachia* strains after culture with its infected host (*Drosophila simulans*) (Heath *et al.*, 1999). Experimental transmission of *Wolbachia* from infected hosts to non-infected eggs by microinjection of egg cytoplasm was obtained, for example, between closely related host species, *Drosophila simulans* and *D. melanogaster* (Boyle *et al.*, 1993), between flies of different genera, *Rhagoletis cerasi* and *Ceratitis capitata* (Zabalou *et al.*, 2004), and between species of different families, like *Drosophila simulans* and *Aedes albopictus* (Braig *et al.*, 1994).

The large variability of *Wolbachia* strains, either in single or multiple infection cases, may also be due to the appearance of distinct haplotypes by recombination events. Putative recombinant haplotypes involving distinct *Wolbachia* strains were found to be widespread among insect species (Jiggins *et al.*, 2001; Werren and Bartos, 2001; Reuter and Keller, 2003; Baldo *et al.*, 2005, 2006a; Arthofer *et al.*, 2009; Yang *et al.*, 2012, 2013). Intragenic recombination occurs frequently in the *wsp* gene of *Wolbachia,* infecting a large number of insect species (Baldo *et al.*, 2005, 2006a). This gene is highly variable and, for this reason, not reliable for phylogenetic inferences, but it is useful for identifying groups of closely related alleles (Baldo and Werren, 2007). The high variability is not distributed evenly along the gene: there are four hypervariable regions (HVR) that are isolated from each other by conserved regions (CR) (Baldo *et al.*, 2005). The portions of the Wsp protein coded by the HVRs form loops outside the bacteria cell and are assumed to participate in establishing the relationships of the bacteria with their hosts. Actually, new Wsp proteins are due largely to mutation, but recombination seems to account for 50% of amino acid differences in recent diverged proteins (Baldo *et al.*, 2010).

Among the frugivorous tephritid flies, *Wolbachia* was found to infect species of the genera *Rhagoletis* (Riegler and Stauffer, 2002; Schuler *et al.*, 2009, 2011, 2013; Arthofer *et al.*, 2009; Drosopoulou *et al.*, 2011; Augustinos *et al.*, 2014), *Bactrocera* (Kittayapong *et al.*, 2000; Jamnongluk *et al.*, 2002; Liu *et al.*, 2006; Sun *et al.*, 2007; Morrow *et al.*, 2014, 2015), *Dacus* (Kittayapong *et al.*, 2000), *Ceratitis* (Rocha *et al.*, 2005), and *Anastrepha* (Werren *et al.*, 1995; Selivon *et al.*, 2002; Coscrato, *et al.*, 2009; Cáceres *et al.*, 2009; Marcon *et al.*, 2011; Martínez *et al.*, 2012). Like in other cases of *Wolbachia* infections, a non-congruence of the endosymbiont phylogenies and their hosts was also observed in fruit flies, suggesting the occurrence of horizontal transmission events (Jamnongluk *et al.*, 2002; Sun *et al.*, 2007; Coscrato *et al.*, 2009). Another way of horizontal transfer of the bacteria among fruit flies would be through the common association of fruit flies with parasitoids, as suggested for species of *Bactrocera* and parasitoid wasps of the genus *Fopius* (Morrow *et al.*, 2014).

Parasitoid wasps of the families Braconidae, Figitidae (Eucolinae) and Pteromalidae have a worldwide distribution (O'Neill *et al.*, 1992; Godfray, 1994), and in the Brazilian territory they are largely dispersed, using as hosts several insect species including *Anastrepha* (Canal and Zucchi, 2000). Although the Braconidae encompass the largest number of species that use fruit flies as hosts (Leonel Jr *et al.*, 1995; Ovruski *et al.*, 2000; Marinho *et al.*, 2009), no studies about *Wolbachia* infection in these fruit fly-parasitoid communities were found. The present report describes the results of an analysis of *Wolbachia* infection involving communities of eight species of *Anastrepha* and six species of braconid wasps derived from these fly hosts. The data show: (a) a very large species infection rate in both insect groups, (b) that several species of wasps share identical *Wolbachia wsp* alleles with distinct species of their *Anastrepha* hosts, and (c) signatures of recombination between *wsp* alleles. The data indicate that horizontal transmission of the *wsp* gene may have occurred in guilds of fruit fly-parasitoids.

## Materials and Methods

### Collection of infested fruits

The species of fruit flies and the associated braconid parasitoids used in the present study derived from infested fruits collected in several locations in Brazil (Table 1 and Figure S1). The localities of collection were chosen in order to collect fruits known, in most cases, to host single species of *Anastrepha*. For example, after several collections only *Anastrepha obliqua* was recovered from starfruit from the city of Indaiatuba. The fruits brought to the laboratory were divided into small groups, which were kept under standard conditions until emergence of adult flies. The emerged adult females of both fruit flies and parasitoids were fixed in 100% ethanol and stored at −20 °C. Identification of fruit flies and braconid wasp species was made according to established criteria (Canal and Zucchi, 2000; Zucchi, 2007; Selivon *et al.*, 2004, 2005).

**Table 1:** Collection sites host fruits and recovered species of *Anastrepha* and of associated braconid wasps.

Collection sites	Host fruits	*Anastrepha*	wasps
São Paulo-SP	“pombeiro”	*amita*	*D. areolatus*
23°32′S / 46°37′W	*Citharexylum myriantum*		*D. brasiliensis*
			*O. bellus*
			*U. anastrephae*
São Paulo-SP	Guava	*fraterculus* sp.1	*D. areolatus*
23°32′S / 46°37′W	*Psidium guajava*		*D. brasiliensis*
			*O. bellus*
			*U. anastrephae*
Vargem Grande-SP	Japanese plum	*fraterculus* sp.1	*D. brasiliensis*
23°39′S /46°59′W	*Eriobotrya japonica*		
Indaiatuba-SP	star fruit	*obliqua*	*A. anastrepha*
23°05′S / 47°13′W	*Averrhoa carambola*		*D. areolatus*
			*O. bellus*
			*U. anastrephae*
Boiçucanga-SP	tropical almond	*fraterculus* sp.2	*D. areolatus*
23°47′S/ 45°37′W	*Terminalia catappa*		*O. bellus*
			*U. anastrephae*
Caçapava-SP	star fruit	*obliqua*	*A. anastrephae*
22°57′S / 48°11′W	*Averrhoa carambola*		
Taubate-SP	manihot	*montei*	*D. fluminensis*
22°57′S / 45°38′W	*Manihot esculenta*	*pickeli*	*D. fluminensis*
Lorena-SP	mango	*obliqua*	*U. anastrephae*
22°44′S / 45°06′W	*Mangifera indica*		
Bemposta-RJ	mango	*obliqua*	*D. areolatus*
22°07′S / 43°05′W	*Mangifera indica*		*U. anastrephae*
Brasília-DF	star fruit	*obliqua*	*D. areolatus*
15°47′S / 47°55′W	*Averrhoa carambola*		*O. bellus*
			*U. anastrephae*
Natal-RN	“burra leiteira”	*macrura*	*D. areolatus*
05°48′S / 35°13′W	*Ficus organensis*	*serpentina*	*D. areolatus*

### DNA extraction and amplification

DNA was extracted from abdomens of females (Jowett, 1986). For the fruit flies, abdomens from three to seven flies were individually analyzed per species and sample. For the braconids, three to four abdomens were pooled for each extraction, and three to nine extractions were made for samples of each species. Amplification was done using primers for the *Wolbachia wsp* gene (Zhou *et al.*, 1998), *wsp* 81F (5TGGTCCAATAGTGATGAAGAAAC3) and *wsp* 691R (5AAAAATTAAACGCTACTCCA3). The PCR reaction consisted of a 3 μL of the extracted DNA of each sample, 2 μL of 10 buffer (Invitrogen), 1.0 μL of MgCl_2_ (50 mM), 1.0 μL of nucleotide mix (5 mM each), 0.5 μL of forward and reverse primers (20 μM each), 1 U of *Taq* DNA polymerase (Invitrogen), and distilled deionized water to a final volume of 20 μL. The amplification cycle was as follows: one cycle (2 min at 95 °C), 35 cycles (1 min at 95 ° C, 1 min at 55 ° C, two2 min at 75 °C), and an extension of 5 min at 72 ° C (Werren *et al.*, 1995). For electrophoresis, 5 μL of each PCR product were run on a 0.8% gel to determine the presence and size of the amplified DNA fragments. About 15% of the PCR products were electrophoresed in 0.8% agarose gel (Gibco) in horizontal system and TAE 1X buffer (40 mM Tris-acetate; 1 mM EDTA, pH 8.0) at 86 V. The samples were mixed with 0.015% bromophenol blue, 0.015% of xylene cyanol and 30% of glycerol (20% in buffer). The DNA fragments were visualized after staining with 5 μg/mL ethidium bromide in a UV transilluminator. Samples of *Wolbachia*-infected *Ceratitis capitata* (Rocha *et al.*, 2005) were used as a positive control for the PCR assays. In case of a negative amplification, the sample DNA was tested for amplification of the 28S rDNA using the universal arthropod primers, and samples that were negative were discarded only after changing the DNA concentrations and PCR conditions (Werren *et al.*, 1995). In case of negative results, new DNA extractions from individuals of that sample were made and the procedure repeated as described above.

### Sequencing and cloning

Fragments of the expected size (~650 bp) were excised from the agarose gels using a purification kit (MagSep Tissue gDNA, Eppendorf) according to the manufacturer's instructions, and these were then sequenced using the BigDye reaction kit (Applied Biosystems) in an ABI-377 Prism automatic sequencer (Applied Biosystems). Sequence reactions were repeated until at least two replicates of the extremities of each sequence were obtained. The electropherograms were examined by the web tool Electropherogram Quality Analysis (Togawa and Brigido, 2003). Beside these analyses, sequences without signals of PCR artifacts were considered free of errors if they were found in more than two individuals in a given sample, and for unique sequences if their amino acid conceptual translation was achieved without interruptions (Yang *et al.*, 2012). For sequences with evidence of two distinct nucleotides in any given peak in the electropherogram, the amplified fragments were cloned in Top 10 *E. coli* bacteria using the Topo Cloning kit (Invitrogen). Bacteria were grown in 3 mL of LB culture medium containing 100 μg/mL of Carbemicillin, and incubated overnight at 37 ° C under rotation at 200 rpm. From the cultures, 1.5 mL was transferred to a polypropylene tube, and centrifuged at 20,800 g for 1 min at room temperature. The pellet was suspended in 100 mL of GTE (20 mM Tris, 50 mM glucose, 10 mM EDTA, pH 8.0) to which 200 μL of 0.2 N NaOH, 1% SDS was added and homogenized by inversion. After addition of 150 μL of 3 M sodium acetate (pH 4.8), centrifugation at 20,800 g for 6 min, the upper layer was transferred to another tube, pure ethanol was added to a 1.5 mL final volume and the tube shacken vigorously. After centrifugation, the pellet was washed with 70% ethanol, dried at 37 °C, and suspended in 50 μL of TE buffer containing 20 μg/mL of RNase A (Sigma). Ten clones of each sample were sequenced using the primers included in the cloning kit. The sequences are available at the *Wolbachia* WSP database and may be assessed by their allele codes.

### Sequence analysis

The sequences were aligned using the Clustal Omega program (Sievers *et al.*, 2011). Identification of haplotypes was made by the DnaSP 5.10 software (Librado and Rozas, 2009), and the distance matrices between sequences of the *wsp* gene were generated in MEGA 6 software (Tamura *et al.*, 2013). The sequences were submitted to the WSP Typing methodology to determine the existing WSP alleles. This is based on the independent variability of the four hypervariable regions and half of each conserved region (HVR**+)**. The alleles are defined by four numerical codes and each identifies one of the HVR**+** regions (Baldo *et al.*, 2005). The HVR profiles were compared to sequences in the *Wolbachia* WSP database and those that had no matches were submitted to the *Wolbachia* database curators for inclusion as new alleles. Occurrence of *wsp* alleles in *Wolbachia* found in fruit flies and in wasp species was assessed by a chi-square test in a contingency table. Alleles found in low frequency (n < 2) could not be included in these tests (Stahlhut *et al.*, 2010). Search for signatures of recombination was made by comparison among the HVR amino acid motifs (Baldo *et al.*, 2005) and by three statistical methods: Maxchi (Maynard Smith, 1992), Geneconv (Posada and Crandall, 2001) and Chimaera (Padidam *et al.*, 1999), implemented in the RDP3.10 software (Heath *et al.*, 2006). In these tests, parameters previously used in analyses of other insects were employed (Baldo *et al.*, 2006b).

## Results and Discussion

### Recovered species of fruit flies and wasps

From the 11 samples of infested fruits, eight species of *Anastrepha* were recovered: *A. amita, A. macrura, A. montei, A. obliqua, A. pickeli, A. serpentina, A.* sp.1 *aff. fraterculus* and *A.* sp.2 *aff. fraterculus* (Table 1). Among the braconid wasps, six species were recovered: *Doryctobracon areolatus, Doryctobracon brasiliensis, Doryctobracon fluminensis, Opius bellus, Utetes anastrephae,* and *Asobara anastrephae* (Table 1). Table 1 also shows the associations of the six wasp species with their *Anastrepha* hosts. The species of braconid wasps that were recovered confirm previous observations that they are commonly dispersed in southeastern Brazil (Canal and Zucchi, 2000; Marinho *et al.*, 2009). An *Anastrepha* species not usually found in southern regions (*Anastrepha macrura*) was collected in a sample from the northeastern city of Natal (Zucchi, 2007).

### Detection and characterization of *Wolbachia wsp* alleles

Out of 62 females of eight species of *Anastrepha* individually screened for *Wolbachia*, 58 turned out to be infected. The sample of *A. serpentina* was the only uninfected one. One hundred and twenty-four out of 140 samples of the six species of braconid wasps, each composed of pooled individuals, were positive for *Wolbachia*, while two samples of *Asobara anastrephae* and one sample of *O. bellus* were not infected. However, a sample of *A. serpentina* from southeastern Brazil was previously found to host a strain of *Wolbachia* (Coscrato *et al.*, 2009). The only other case of *Wolbachia*-free *Anastrepha* was found in samples of *A. ludens* from Mexico (Martínez *et al.*, 2012). From the *Anastrepha* species screened so far, 14 out of 15 (93.3%) were infected by *Wolbachia* (Werren *et al.*, 1995; Selivon *et al.*, 2002; Coscrato *et al.*, 2009; Cáceres *et al.*, 2009; Martínez *et al.*, 2012). This is a very high infection rate even among tephritid flies since, for example, in *Bactrocera* from Thailand *Wolbachia* infection occurred in 28.3% of the species (Kittayapong *et al.*, 2000) and in 37% of species of fruit flies, including *Bactrocera* from Australia (Morrow *et al.*, 2015). Amongst the braconids, five out of six (83.3%) species were infected by the endosymbiont, a rate similar to the 84% (14 out of 17 species) found in fig wasps from China (Yang *et al.*, 2012). Thus, the species infection rate found in *Anastrepha* and in the parasitoid wasps are among the highest found in insect species which span from 40 to 76% (Werren and Windsor, 2000; Jeyaprakash and Hoy, 2000; Hilgenboecker *et al.*, 2008; Zug and Hammerstein, 2012).

In every species and samples of fruit flies and wasps, local alignment (BLASTN) of the sequences to the WSP database showed that the amplified fragments were from the *wsp* gene of supergroup A *Wolbachia.* Species of the *Bactrocera* and *Rhagoletis* fruit flies harbor *Wolbachia* strains of groups A and B (Jamnongluk *et al.*, 2002; Sun *et al.*, 2007; Arthofer *et al.*, 2011), but in *Anastrepha,* group B was so far found only in *A. striata* from Mexico (Martínez *et al.*, 2012), and in a sample of unknown origin of nominal *A. fraterculus* (Cáceres *et al.*, 2009). In line with previous data, infection by *Wolbachia* supergroup A is prevalent among distinct host insects, including the Diptera and Hymenoptera (Werren *et al.*, 1995; Stahlhut *et al.*, 2010; Baldo *et al.*, 2010).

Among the entire set of nucleotide sequences, regardless of whether they were from the fruit flies or the braconids, DnaSP detected 22 *wsp* nucleotide haplotypes. Assuming that the distinctiveness of *Wolbachia* haplotypes is recognized just for those differing in more than 1.5% (Zhou *et al.*, 1998; Zabalou *et al.*, 2004; Sintupachee *et al.*, 2006), the 22 haplotypes formed eight groups, named as w1, w2, w3, w4, w5, w6, w7 and w8. The intragroup distance varied from 0.002 (w8) to 0.007 (w1), and the intergroup distances varied from 0.022 (w4/w7) to 0.258 (w1/w7) (Table S1). The sequences were further analyzed by the WSP Typing methodology (Baldo *et al.*, 2005) that, based on the four HVR peptides, detected eight *wsp* alleles of *Wolbachia* infecting the guilds of fruit flies and braconid wasps (Table 2). These *wsp* alleles correspond to the eight haplotypes determined by the nucleotide sequences: wsp-75 (w8), wsp-23 (w3), wsp-156 (w1), wsp-680 (w2), wsp-681(w4), wsp-682 (w5), wsp-683 (w6) and wsp-684 (w7). Three WSP alleles, wsp-23, wsp-75 and wsp-156, were found in the WSP database and occur in *Wolbachia* infecting a variety of insect species (Baldo *et al.*, 2005, 2010). The other five alleles, wsp-680, wsp-681, wsp-682, wsp-683 and wsp-684, are novel *wsp* alleles detected in the present analysis.

**Table 2:** Wsp HVR profiles of *Wolbachia* infecting *Anastrepha* and parasitoid braconid wasps.

Haplotype groups	Peptide codes				WSP alleles
	HVR1+	HVR2+	HVR3+	HVR4+	
w1.0	71	34	15	25	156
w2.0	235	15	17	14	680
w3.0	1	12	21	19	23
w4.0	1	12	265	14	681
w5.0	236	12	21	19	682
w6.0	1	15	17	14	683
w7.0	232	12	266	14	684
w8.0	11	9	15	25	75

The present data show that the number of different *wsp* alleles of *Wolbachia* infecting *Anastrepha* species is higher than found in a previous screening based on nucleotide haplotypes of 10 species, in which the sequences were very similar to wMel (Coscrato *et al.*, 2009). The high rate of species infection can be explained by assuming that the fruit flies and parasitoid wasps may be highly prone to infection by the bacteria, and may be favored by the combination of their habitats and life strategies, allowing horizontal transmissions, in line with observations in other insect species (Werren *et al.*, 2008; Stahlhut *et al.*, 2010). These facts may also be a possible explanation for infection by multiple *Wolbachia* strains found in many hosts insects (Werren *et al.*, 1995; Rokas *et al.*, 2002; Kittayapong *et al.*, 2003; Reuter and Keller, 2003; Hiroki *et al.*, 2004; Yang *et al.*, 2013).

### Wsp alleles in fruit flies and parasitoid wasps

Table 3 summarizes the *Wolbachia* harboring distinct *wsp* alleles found in each species and sample of *Anastrepha,* as well as in the braconids that emerged from the puparia of the sampled host fruit flies. The present analysis showed that most *Anastrepha* species were infected by a single *Wolbachia* strain, but a double infection (sample from Indaiatuba) and a multiple infection (sample from Caçapava) were found in *A. obliqua.* Similarly, two *Wolbachia* bearing distinct alleles were found only in a sample (from São Paulo) of the parasitoid *D. brasiliensis,* while a single infection was found in the other five wasp species. The data in Table 4 show significant differences (X^2^ = 33.13, d.f. = 2, P < 0.001) in the distribution of *Wolbachia* harboring distinct *wsp* alleles between the fruit flies and the wasps. *Wolbachia* wsp-23 was more frequent in flies (76.3%) than in wasps, while *Wolbachia* wsp-156 and *Wolbachia* wsp-680 were more frequent in wasps than in the fruit flies hosts (80.9% and 94.1%, respectively). In the majority of cases, *Wolbachia* infecting the fruit flies were distinct from those detected in the braconid wasps with respect to the *wsp* alleles. However, in some samples, *Wolbachia* infecting the fruit flies had an identical *wsp* allele as the bacteria infecting the parasitoid wasps. Table 5 shows the congruence involving *Wolbachia* bearing allele wsp-23 or allele wsp-156 between fruit flies and wasps. The striking cases seem to be those of *Wolbachia* wsp-156 found in *D. fluminensis* derived from two species of fruit flies, and of *Wolbachia* wsp-23 that was found in *Utetes anastrephae* derived from four species of *Anastrepha*. These data indicate that horizontal transmission might have occurred in the communities of fruit flies and braconid wasps, similarly to what was assumed in guilds of other insects with their parasitoid wasps (Vavre *et al.*, 1999; Yang *et al.*, 2012; Yang *et al.*, 2013; Morrow *et al.*, 2014).

**Table 3:** *Wolbachia* alleles* in species of *Anastrepha* associated with braconid parasitoids, localities of collection and number of samples (N) screened for *Wolbachia.*

*Anastrepha*			Braconid wasps			Samples
Species	*Wolbachia*	N^a^	Species	*Wolbachia*	N^b^	
***amita***	**wsp-23**	5	*D. areolatus*	wsp-680	6	São Paulo-SP
			*D. brasiliensis*	wsp-75, −156	6	
			*O. bellus*	not infected	6	
			*U. anastrephae*	**wsp-23**	**5**	
*fraterculus*	wsp-23	4	*D. brasiliensis*	wsp-156	4	Vargem Grande-SP
(sp.1)	**wsp-23**	7	*D. areolatus*	wsp-680	6	São Paulo-SP
			*D. brasiliensis*	wsp-156	4	
			*O. bellus*	wsp-680	6	
			*U. anastrephae*	**wsp-23**	**5**	
*fraterculus*	**wsp-23**	6	*D. areolatus*	wsp-680	6	Boiçucanga-SP
(sp.2)			*O. bellus*	wsp-680	5	
			*U. anastrephae*	**wsp-23**	**6**	
*macrura*	**wsp-156**	4	*D. areolatus*	**wsp-156**	**4**	Natal-RN
*serpentina*	not infected	4	*D. areolatus*	wsp-156	4	
*montei*	**wsp-156**	4	*D. fluminensis*	**wsp-156**	**4**	Taubaté-SP
*pickeli*	**wsp-156**	4	*D. fluminensis*	**wsp-156**	**4**	
*obliqua*	**wsp-23, -**684	6	*A. anastrephae*	not infected	4	Indaiatuba-SP
			*D. areolatus*	wsp-156	6	
			*O. bellus*	**wsp-23**	**6**	
			*U. anastrephae*	**wsp-23**	**6**	
	wsp-23	4	*D. areolatus*	wsp-156	6	Lorena-SP
	**wsp-23**	4	*D. areolatus*	wsp-156	6	Bemposta-RJ
			*U. anastrephae*	**wsp-23**	**6**	
	**wsp-23**	4	*D. areolatus*	wsp-156	5	Brasília-DF
			*O. bellus*	wsp-680	3	
			*U. anastrephae*	**wsp-23**	**5**	
	wsp-23, −680,	6	*A. anastrephae*	not infected	6	Caçapava-SP
	−681, −682, −683					

* In bold, congruence of *Wolbachia* alleles in fruit flies and associated wasps

N^a^: number of *Anastrepha* females individually screened. N^b^: number of screened samples of wasps each composed of 3-4 pooled females.

**Table 4:** Distribution of *Wolbachia* bearing distinct alleles in the fruit flies and wasps.

Species	wsp alleles*				Test	P-value
	23	156	680	Total		
Fruit flies	45	12	2	59	X^2^ = 33.13	< 0.001
Braconid wasps	39	51	32	122		
Total	84	63	34	181		

* Alleles wsp-75 and the recombinants were not included due to insufficient numbers.

**Table 5:** Congruence of *Wolbachia* infecting species of braconid wasps and their *Anastrepha* host species.

Braconids		Allelic association flies//braconids			
species	samples	N	%	alleles	*Anastrepha* host
*D. areolatus*	8	1	0.125	wsp-156	*macrura*
*D. fluminensis*	2	2	1.000	wsp-156	*montei; pickeli*
*O. bellus*	4	1	0.250	wsp-23	*obliqua*
*U. anastrephae*	6	6	1.000	wsp-23	*amita; fraterculus-1 fraterculus 2; obliqua*

### Recombination between *wsp* alleles

The presence of two or more *wsp* sequences in single individuals offers an opportunity for the appearance of new haplotypes through events of recombination, which consequently contributes to the increase in the number of *Wolbachia* variants. Recombination between *Wolbachia* sequences is widespread among insects, and the recombinant haplotypes are assumed to be functional (Reuter and Keller, 2003; Baldo *et al.*, 2005, 2006a,b, 2010; Baldo and Werren, 2007). Those involving the *wsp* gene seem to produce novel phenotypes that could create new possibilities for the bacteria to explore new hosts (Werren *et al.*, 2008; Baldo *et al.*, 2010).

**Figure 1:**
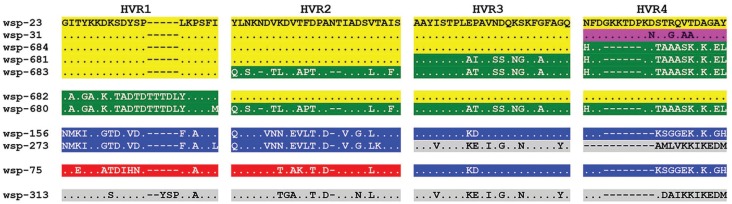
Amino acid motifs of the hypervariable regions (HVRs) of *Wolbachia wsp* alleles infecting species of *Anastrepha* and associated parasitoid braconid wasps. The sequences were aligned relative to the wsp-23 allele. The intervening conserved regions (CR) were omitted from the sequences. The HVR motifs were grouped according to similarity of polymorphism and taking HVR1 as the reference for grouping. Each *wsp* allele has a unique combination of HVRs indicated by colors, which are interpreted as the result of HVR shuffling.

In the present study, a search for recombination signatures within the communities of fruit flies-parasitoids was made by analysis of the four HVR amino acid motifs according to Baldo *et al.* (2005). For these analyses, besides the three alleles previously known, wsp-23, wsp-75 and wsp-156, the WSP database was searched for *wsp* alleles that would have sequences partially similar to the novel five alleles herein detected. Three *Wolbachia* alleles with high similarity were found: wsp-31 from *Wolbachia* infecting *Drosophila melanogaster* host, and two from ant species hosts, wsp-273 from *Formica truncorum* and wsp-313 from *Formica exsecta* hosts. Since wsp-23 is considered an ancestral *Wolbachia wsp* allele (Baldo *et al.*, 2005, 2010), its amino acid sequence was taken as reference for the present analysis (Figure 1). The previous known alleles, wsp-75 and wsp-156, differed from wsp-23 in their four HVRs (Table 2). As known, wsp-31 from *Wolbachia* wMel is considered a recombinant sequence differing from wsp-23 in HVR4 (Baldo *et al.*, 2005). The novel wsp-680 has its four HVRs distinct from those of wsp-23. Signals of HVR shuffling were found for the other four new alleles: (a) wsp-681 might be a recombinant allele since it has HVR1 and HVR2 identical to those of wsp-23, but HVR3 and HVR4 identical to the corresponding ones in wsp-680 and wsp-682; (b) wsp-683 and wsp-684 would be recombinants involving distinct HVRs between wsp-23 and wsp-680. The alleles wsp-75 and wsp-156 seem to be involved in recombination with alleles wsp-273 and wsp-313, both from ant species.

**Figure 2:**
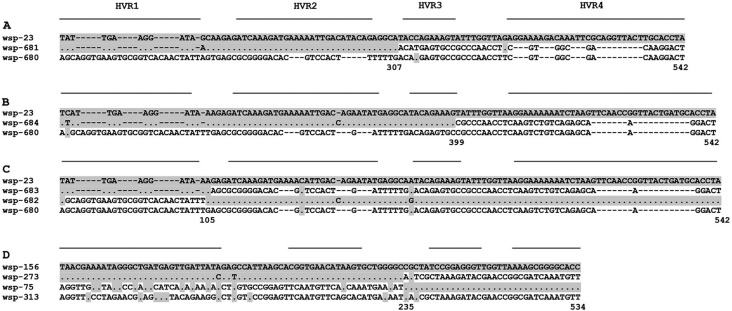
Putative recombination detected among *Wolbachia wsp* alleles infecting species of *Anastrepha* and associated parasitoid braconid wasps. In each alignment, only the polymorphic sites of the sequences are shown. Gray shaded parts of sequences are polymorphisms shared with the top sequence in each alignment. Sequences in the middle of each alignment were indicated as recombinant sequences, and the top and bottom sequences as the two parental sequences. The numbers below the alignments indicate the approximate nucleotide position of the breakpoints detected by three methods (Maxchi, Geneconv, Chimaera). The lines above the sequences indicate the position of the four HVRs.

Moreover, signatures of recombination were tested by three statistical methods, Maxchi, Geneconv and Chimaera, and only the putative events concomitantly disclosed by the three methods were considered. Figure 2 shows the results of this analysis and the three methods gave very significant P values (P < 0.000001) for every case tested. The data confirmed the visual analysis made on the HVR amino acid motifs described above, and showed that wsp-681 (Figure 2A) and wsp-684 (Figure 2B) may represent distinct recombinant haplotypes between wsp-23 and wsp-680. Two other cases were found involving four sequences with a single breakpoint each. As shown in Figure 2C, besides the parental sequences (wsp-23 and wsp-680), two putative recombinant sequences were found (wsp-682 and wsp-683), and, shown in Figure 2D, two parental sequences (wsp-156 and wsp-313) and two possible reciprocal recombinants (wsp-75 and wsp-273) were found. The origin of these reciprocal recombinant sequences could be due to independent events of recombination or to reciprocal exchange of single events, as discussed previously for putative recombination in other insects (Baldo *et al.*, 2005). In every case of recombination the breakpoints occurred in the limits of the HVRs and the CRs intervening regions, as was usually described for *wsp* recombination in other insects (Baldo *et al.*, 2005).

Signatures of intragenic recombination of the *wsp* gene, detected for the first time in *Anastrepha* hosts in the present analysis, were found in *Wolbachia* infecting *Anastrepha obliqua*. The other case of inferred putative recombination involved two alleles, wsp-75 and wsp-156, found in the parasitoid *Doryctobracon brasiliensis,* with *wsp* sequences of *Wolbachia* previously found in two ant species, allele wsp-273, from *Formica truncorum* host, and wsp-313 from *F. exsecta* host. The way these putative recombinant events have occurred is unknown, but it should involve the presence of different *Wolbachia* strains in the fruit flies and/or wasps and in the ant species. Evidence of interspecies transfer of *Wolbachia* was found previously in the social parasitism of two ant species (*Solenopsis* spp) with parasitoids and a social parasite (Dedeine *et al.*, 2005). In this scenario, besides the close ecological relationships between fruit flies and parasitoid wasps, one may assume that they also share ecological proximity to ants. Indeed, fruit fly species have ants as one of their most important predators during the life stages when they are exposed in soil, as mature larvae when they leave the fruits, as pupae and as emerging adults (Bateman, 1992). Hence, predation of *Anastrepha* by ants infected with *Wolbachia* and carrying eggs of parasitoid wasps, may be a possible way of horizontal transmission of *Wolbachia* between these three insect clades, and could account for the suggestive recombination events herein described.

Recombination between *Wolbachia* haplotypes seems infrequent among fruit fly hosts. Strain wCer3 of *Rhagoletis* has been suggested to be a recombinant between A and B *Wolbachia* supergroups (Arthofer *et al.*, 2009), and no recombinants were yet described in the genus *Bactrocera* (Kittayapong *et al.*, 2000; Jamnongluk *et al.*, 2002; Sun *et al.*, 2007; Morrow *et al.*, 2014, 2015). Our data indicate that a similar situation seems to occur for *Wolbachia* infecting *Anastrepha* species.

## Concluding Remarks

The present analysis shows a high infection rate for fruit flies and braconid wasps and the occurrence of putative intragenic recombination between *Wolbachia wsp* sequences. By screening for *Wolbachia* infection in *Anastrepha* species and in braconid wasps that emerged from samples of these fly species we obtained for the first time strong evidence for horizontal transmission between these two groups of insects. Horizontal transmission also explains the widespread occurrence of *Wolbachia* bearing a given *wsp* allele, as is known for a large number of insect species (Baldo *et al.*, 2010; Stahlhut *et al.*, 2010). One such case is the ancestral allele wsp-23 detected in *Wolbachia* from at least 21 species, 11 genera and 11 families, but found preferentially in Diptera and Hymenoptera (Baldo *et al.*, 2010). *Wolbachia* bearing this allele was found also in tephritid flies, in species of *Anastrepha* (Coscrato *et al.*, 2009), *Rhagoletis cerasi* (wCer2) (Arthofer *et al.*, 2011), *R. pomonella* (wPom1) (Schuler *et al.*, 2011), and in the fly-wasp guilds, studied herein. Since *Wolbachia* strains usually do not persist for long periods of time in a given host (Baldo *et al.*, 2008, 2010), the most parsimonious hypothesis to explain the presence of *Wolbachia* wsp-23 in *Rhagoletis*, in *Anastrepha* and in the parasitoids found, might be by horizontal transmission.

## Supplementary Material

The following online material is available for this article:

Figure S1 - Approximate locations where infested fruits were collected in Brazil.

Table S1 - Genetic distances among *wsp* nucleotide haplotypes of *Wolbachia*.
