# Supplementary material for: Wolbachia in guilds of Anastrepha fruit flies (Tephritidae) and parasitoid wasps (Braconidae)
Source: Genet Mol Biol. 2016 Sep 19;39(4):600–10. doi: 10.1590/1678-4685-GMB-2016-0075 (PMC5127160; doi:10.1590/1678-4685-GMB-2016-0075)
Supplement: Supplementary file 1 [file 1415-4757-gmb-1678-4685-GMB-2016-0075-Suppl02.pdf]

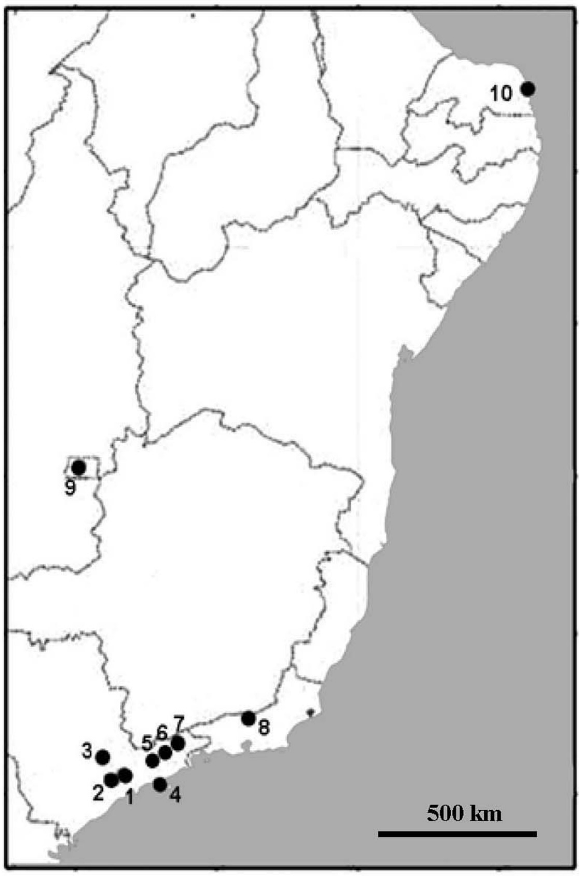

Figure S1. Approximate location of collection in Brazil:
1. São Paulo-SP; 2. Vargem Grande-SP; 3. Indaiatuba-SP;
4. Boiçucanca-SP; 5. Caçapava-SP; 6. Taubaté-SP;
7. Lorena-SP; 8. Bemposta-RJ; 9. Brasília-DF; 10. Natal-RN.

Figure S1 - Approximate locations where infested fruits were collected in Brazil: 1. São Paulo-SP; 2. Vargem Grande-SP; 3. Indaiatuba-SP; 4. Boiçucanga-SP; 5. Caçapava-SP; 6.Taubate-SP; 7. Lorena-SP; 8. Bemposta-RJ; 9. Brasília-DF; 10. Natal-RN.
