# Supplementary material for: Wolbachia in guilds of Anastrepha fruit flies (Tephritidae) and parasitoid wasps (Braconidae)
Source: Genet Mol Biol. 2016 Sep 19;39(4):600–10. doi: 10.1590/1678-4685-GMB-2016-0075 (PMC5127160; doi:10.1590/1678-4685-GMB-2016-0075)
Supplement: Supplementary file 2 [file 1415-4757-gmb-1678-4685-GMB-2016-0075-Suppl01.pdf]

Table S1 - Genetic distances among *wsp* haplotypes of *Wolbachia* in species of *Anastrepha* and braconid wasps.

	w1.0	w1.1	w1.2	w1.3	w1.4	w1.5	w1.6	w1.7	w1.8	w2.0	w3.0	w3.1	w3.2	w3.3	w.4.0	w4.1	w5.0	w6.0	w7.0	w8.0
w1.0																				
w1.1	0.002																			
w1.2	0.004	0.006																		
w1.3	0.002	0.004	0.006																	
w1.4	0.006	0.008	0.009	0.008																
w1.5	0.006	0.007	0.009	0.007	0.011															
w1.6	0.004	0.006	0.007	0.006	0.009	0.009														
w1.7	0.004	0.006	0.008	0.006	0.009	0.002	0.007													
w1.8	0.004	0.006	0.007	0.006	0.009	0.009	0.008	0.007												
w2.0	0.236	0.240	0.233	0.240	0.236	0.246	0.236	0.243	0.240											
w3.0	0.202	0.206	0.197	0.206	0.206	0.211	0.206	0.208	0.206	0.208										
w3.1	0.202	0.206	0.197	0.206	0.206	0.211	0.206	0.208	0.206	0.208	0.000									
w3.2	0.209	0.212	0.203	0.212	0.212	0.218	0.212	0.215	0.212	0.215	0.004	0.004								
w3.3	0.205	0.209	0.200	0.209	0.209	0.214	0.209	0.211	0.209	0.211	0.002	0.002	0.006							
w4.0	0.261	0.265	0.257	0.265	0.257	0.271	0.261	0.267	0.265	0.099	0.088	0.088	0.092	0.090						
w4.1	0.269	0.273	0.265	0.273	0.265	0.278	0.269	0.275	0.273	0.102	0.090	0.090	0.095	0.092	0.006					
w5.0	0.216	0.219	0.210	0.219	0.219	0.225	0.219	0.222	0.219	0.165	0.033	0.033	0.037	0.035	0.129	0.132				
w6.0	0.222	0.225	0.219	0.225	0.222	0.231	0.222	0.228	0.225	0.035	0.156	0.156	0.162	0.159	0.058	0.060	0.205			
w7.0	0.237	0.240	0.233	0.240	0.233	0.246	0.237	0.243	0.240	0.121	0.067	0.067	0.072	0.069	0.021	0.023	0.107	0.078		
w8.0	0.106	0.109	0.111	0.109	0.109	0.114	0.111	0.111	0.109	0.252	0.127	0.127	0.132	0.129	0.175	0.181	0.161	0.213	0.155	
w8.1	0.109	0.111	0.114	0.111	0.111	0.116	0.114	0.114	0.111	0.255	0.129	0.129	0.135	0.132	0.178	0.184	0.164	0.216	0.158	0.002
